# Liver Regeneration: Different Sub-Populations of Parenchymal Cells at Play Choreographed by an Injury-Specific Microenvironment

**DOI:** 10.3390/ijms19124115

**Published:** 2018-12-18

**Authors:** Rita Manco, Isabelle A. Leclercq, Laure-Alix Clerbaux

**Affiliations:** Laboratory of Hepato-Gastroenterology, Institut de Recherche Expérimentale et Clinique, UCLouvain, Avenue E Mounier 53, 1200 Brussels, Belgium; rita.manco@uclouvain.be (R.M.); laure-alix.clerbaux@uclouvain.be (L.-A.C.)

**Keywords:** hepatocytes, liver progenitor cells, ductular reaction, hepatectomy, chronic liver injury, liver failure

## Abstract

Liver regeneration is crucial for the maintenance of liver functional mass during homeostasis and diseases. In a disease context-dependent manner, liver regeneration is contributed to by hepatocytes or progenitor cells. As long as they are replicatively competent, hepatocytes are the main cell type responsible for supporting liver size homeostasisand regeneration. The concept that all hepatocytes within the lobule have the same proliferative capacity but are differentially recruited according to the localization of the wound, or whether a yet to be defined sub-population of hepatocytes supports regeneration is still debated. In a chronically or severely injured liver, hepatocytes may enter a state of replicative senescence. In such conditions, small biliary cells activate and expand, a process called ductular reaction (DR). Work in the last few decades has demonstrated that DR cells can differentiate into hepatocytes and thereby contribute to parenchymal reconstitution. In this study we will review the molecular mechanisms supporting these two processes to determine potential targets that would be amenable for therapeutic manipulation to enhance liver regeneration.

## 1. Introduction

The liver is a vital organ, performing crucial metabolic, synthetic and detoxification functions, such as glucose and lipid metabolism and partitioning, plasma protein and bile acid synthesis, the detoxification of ammonia and the metabolization of xenobiotic agents. A highly organized structure supports the activities of the organ. The lobule is the basic structure of the liver: A hexagonal structure, delimitated by six portal triads, where the structure is centered on a central vein [1]. The portal triad contains a branch of the hepatic artery, a branch of the portal vein and one or two bile duct ramifications. Both the artery and the vein supply blood to the liver, while the bile ducts drain the bile out of the liver. The liver lobule consists of epithelial cells, hepatocytes and cholangiocytes, and of non-parenchymal cells, such as liver sinusoidal endothelial cells (LSEC), Kupffer cells and hepatic stellate cells (HSC). Hepatocytes are the most abundant cells in the liver parenchyma (accounting for 70% of the total liver mass) and represent the metabolically active epithelial cells. They are polarized cells that exhibit three well-defined plasma membrane domains [2]. The basolateral domain faces the sinusoids and is in contact with the space of Disse, the interspace between the hepatocytes and the fenestrated endothelium. This is the site of a bidirectional exchange of molecules between the blood and the hepatocytes. At the lateral domain, tight junctions join adjacent hepatocytes to form the plate. Finally, the biliary/apical domain is the secretory cellular pole at which biliary export pumps and transporters are specifically expressed. The apposition of the edges of biliary domains of two to three adjacent hepatocytes form an intercellular space approximately 1 µm in diameter, called the bile canaliculus, which receives the primary bile. The bile then flows out of the liver through the bile ducts. Cholangiocytes, the cells of the bile duct, are cuboidal in shape and they modulate the composition of bile through the absorption and secretion of ions, solutes and water [3]. The anatomical location where the bile canaliculi and the bile ducts meet is called the Canal of Hering and is the place where biliary/progenitor cells are located [4,5]. The resident liver macrophages, Kupffer cells, are located within the sinusoidal vascular space, where the phagocyte cell debris is brought by the portal stream, and secrete inflammatory factors in response to external stimuli [6]. Finally, the perisinusoidal space of Disse contains the HSCs, vitamin-A-storing cells which are the major fibrogenic cells during injury [7].

The liver has an extraordinary capacity to regenerate, such as after surgical resection of up to 70% of the liver. The residual liver “regrows” to its initial mass in seven days in rodents, or in a few weeks in humans, with no functional loss during the process. This feature of the liver permits large therapeutic surgical resections. In the context of transplantation, the regenerative process adapts the size of the transplanted organ to the receiver’s size, therefore rendering possible liver splitting and living donor transplantation. Recruiting similar processes, regeneration restores liver mass, architecture and function upon acute hepatocellular injury. If the regenerative process is overwhelmed, inefficacious or the cellular damage is too extensive, acute liver failure will ensue. In chronic liver injury, chronic hepatocellular damage constantly stimulates regeneration. An eventual imbalance between the damage and regeneration, or the exhaustion of regeneration, largely contributes to progressive liver insufficiency developing in end-stage liver diseases. Up until to now, liver transplantation represents the only cure for fulminant liver failure and end-stage chronic liver diseases. Given the shortage in organ availability, it is important to seek alternative strategies.

As liver regeneration is a tightly orchestrated process, understanding of the regulatory mechanisms at play is clinically relevant and key for the development of future therapies. The sum of signals which sense the physiologically needed liver mass, and accordingly initiate or terminate liver regeneration, is called the “hepatostat” [8]. Although we now know many signals implicated in liver regeneration, exactly how they interact and integrate to constantly adjust liver mass is less understood. Liver regeneration proceeds through the proliferation of all cell types of the liver, namely the two hepatic epithelial cells (hepatocytes and cholangiocytes) and the non-parenchymal cells (Kupffer cells, hepatic stellate cells and liver sinusoidal endothelial cells). 

## 2. Hepatocyte-Mediated Parenchymal Regeneration

Hepatocytes, which during homeostasis are quiescent cells, undergo several rounds of cell division during regeneration as to repopulate the parenchyma upon partial organ resection. This also occurs in almost all liver diseases. Confirmation for this came from several animal experimental studies in which hepatocytes were specifically tagged using highly efficient adeno-associated viruses 8 (AAV8), showing that after a partial hepatectomy (PH) or an acute carbon tetrachloride (CCl_4_) injury, new hepatocytes derive from the division of pre-existing hepatocytes, with no recruitment of other intra-hepatic or extra-hepatic cells [9,10,11].

### 2.1. Initiating Molecular Signals

The kinetics of liver regeneration mediated by hepatocytes have been mostly studied in PH rodent models, a model that does not cause lobular damage or inflammation [12]. Within minutes after PH [13,14], gene expression in hepatocytes as well as in non-parenchymal cells dramatically changes, resulting in the hepatocytes exiting the G0 phase and entering the cell cycle.

The first mitogenic factor that stimulates hepatocytes is the hepatic growth factor (HGF), normally sequestrated in the extra cellular matrix (ECM) scaffold. Within 5 min after PH, the activity of urokinase (uPA), (which is involved in the cascade that activates the matrix metalloproteinases (MMPs)) increases strongly [12]. This causes a degradation of ECM, inducing the rapid release of matrix-sequestered HGF within one hour. Later on (a timing evaluated as 36 h post partial hepatectomy) hepatic stellate cells (HSCs) and liver sinusoidal endothelial cells (LSECs) produce de novo HGF [15,16], maintaining high levels of active HGF during the regenerative process. Besides HGF and other growth factors, such as the epithelial growth factor (EGF), the inflammatory cytokines Tumor necrosis factor-α (TNFα) and Interleukin-6 (IL-6) play important roles in liver regeneration. Studies have shown that Kupffer cells, which secrete TNF-α and IL-6, are critical for initiating liver regeneration [17]. Mice deficient in TNF-α receptor 1 have low activation of the nuclear factor kappa B (NF-κB) and delayed regenerative response [18]. NF-κB also promotes the production of IL-6. In mice that are genetically deficient in IL-6, hepatocytic proliferation is impaired, resulting in delayed regeneration [19]. On the contrary, IL-6 overexpression accelerates regeneration [20]. 

Another key signal for the regenerative process is the β-catenin pathway. β-catenin translocates from the cytoplasm to the nucleus in hepatocytes 15 min after PH [21], where it controls the transcriptional up-regulation of cyclin-D1. The signaling event is pivotal for the transition from the G1 phase to the S phase of the cell cycle. If mice with a hepatocyte-specific depletion of β-catenin undergo PH, regeneration is substantially delayed, with only half of the hepatocytes in S-phase 40 h after the PH, compared to controls. However, the proliferation rate normalizes 72 h after PH [22,23], suggesting that, although important, β-catenin is not the only driver of entry into the cell cycle. Among other additional important pathways, Notch signaling has an early role in the proliferation of hepatocytes. Although Notch1 depletion in the adult healthy liver without additional injury causes liver hyperplasia, the absence of Notch1 or Jagged-1 (its ligand) delays liver regeneration upon PH [24].

Bile acids (BA) have recently emerged as important signaling modulators of liver regeneration [25]. Upon hepatectomy, the remaining hepatocytes are exposed to high BA levels. BA-activation of nuclear hormone receptors, such as the Farnesoid X receptor (FXR), promotes hepatocyte proliferation via HGF and cyclin-D1 signaling [26]. Conversely, FXR knockout mice display delayed liver regeneration after PH or CCl_4_ injury [27,28]. FXR is proposed to regulate liver size by sensing the organ’s functional capacity reflected by hepatic BA levels. Furthermore, expressed at the cell surface of cholangiocytes and Kupffer cells but not hepatocytes, the BA receptor TGR5 seems to also be required for normal regeneration following PH, supposedly by protecting the liver from BA overload [29,30]. Actually, while BAs are essential for the hepatocytic proliferative process after PH, excessive BA concentrations (too high, too low or too hydrophobic) appear to impair liver regeneration [27,29,30]. This dual regenerative and toxic effect of BAs suggests that a proper BA homeostasis is crucial to ease liver regeneration [31]. Such findings also indicate that the BA pool composition influences the liver regeneration process. BA metabolism by gut microbiota is an understudied potential factor that modulates liver regeneration [32,33].

Finally, it is worth noting that the recovery of initial liver mass, which requires only days to complete in rodents after PH, is not entirely due to cell proliferation, but also to cell hypertrophy. Notably, mass recovery after 30% PH is achieved only by hepatocyte hypertrophy, rather than proliferation [34,35]. In the liver, more than 30% of the hepatocytes are polyploidy; i.e., they contain more than two sets of chromosomes. In rodents, hepatocytes are tetraploid (binuclear with 2 diploid nuclei (2 × 2n) or mononuclear with 1 tetraploid nucleus (4n)) and octoploid (binuclear with 2 tetraploid nuclei (2 × 4n) or mononuclear with 1 octoploid nucleus (8n). In humans, hepatocytes are mostly tetraploid. During regeneration, hepatocyte ploidy increases: After 70% PH, a large proportion of hepatocytes enter the cell cycle, but not all of them complete the cell division. This results in an enlargement of the nuclei and thus hypertrophic polyploid hepatocytes. 

### 2.2. Terminating Molecular Signals

When cell proliferation has restored the right liver/body mass ratio, the regenerative process eventually stops. The mechanisms terminating the process are still largely unknown. Sensing of a recovered functional capacity, including normal hepatic BA levels for example, may interrupt the regenerative process. Signals from ECM also contribute to termination of the process. In vitro studies revealed that when hepatocytes are cultured in an uncoated plastic plate, they have a higher proliferative ratio compared to when grown on ECM-coated wells [36,37,38]. The membrane bound proteoglycan Glypican-3 (GPC3), which is not well expressed in a healthy liver, increases in the termination phase and GPC3 deficient mice have an oversized liver [39]. 

Activation of the Hippo pathway, through its effector Yes-associated protein (YAP), is associated with many cellular processes, including proliferation and organ size. Any modification of the Hippo pathway, specifically in hepatocytes, results in an overgrowth of the liver. Deletion of Mst1/2 (mammalian sterile 20 like kinase), an intermediate in Hippo signaling, results in a significant enlargement of the liver due to unstopped hepatocytic proliferation [40,41] 

Last but not least, TGF-β is a potent inhibitor of hepatocyte proliferation in vitro [42] and in vivo [43]. TGF-β expression decreases after PH and is progressively re-expressed to pre-PH levels at the end of regeneration, probably in link with the restitution of the mass of non-parenchymal cells. However, the lack of a TGF-β II receptor specifically in hepatocytes is not sufficient to impede the termination of regeneration [44]. Additional inhibition of Activin A (another member of the TGF-β family) or blockage of the Activin A receptor is needed to perpetuate cell proliferation beyond normal organ size [44]. 

### 2.3. Hepatocytes Zonation within the Lobule: Not Only for Metabolism

Liver histology shows a homogenous and repetitive pattern of functional lobules. Although morphologically similar, hepatocytes in the lobule are heterogeneous at the metabolic level, and as shown in the most recent publications, they are also heterogeneous at the regenerative level. Metabolic heterogeneity is linked to the position of a cell within the lobular unit and thus exposure to variable blood concentrations in oxygen, substrates and hormones. Cells in zone 1 receive the highest oxygenated blood and are implicated in more energetic processes, such as gluconeogenesis and ureagenesis, while hepatocytes at the center of the lobule (zone 3) which are receiving less oxygen are rather specialized in glycolysis, bile acid synthesis and xenobiotic metabolization [45]. Not only do these functions seem to be location-related in the liver, but also related to the regeneration capacity of the hepatocytes. 

At the end of the 1970s, “the streaming liver” theory [46] hypothesized that during homeostasis there was a constant production of new hepatocytes generated at the outer rim of the portal triad, moving towards the central vein. Autoradiography studies, capturing the DNA synthesis through ^3^H-thymidine incorporation, found 48 h post PH to be the time of the highest percentage of hepatocytes under replication in zone 1, less in zone 2, and few or no labelled hepatocytes around the central vein, zone 3 [47,48]. This was interpreted at that time as confirmation of the streaming liver theory and of the existence of a sub-population of hepatocytes in zone 1, with a high replicative capacity compared to those in the other zones. In 1994 however, Bralet et al. challenged this view. They used a retroviral vector to specifically label and follow the fate of hepatocytes [49]: The pattern of distribution of labelled hepatocytes within the lobule remained constant over 15 months. Hepatocytes located in one zone proliferated and stayed in that specific zone, refuting streaming. 

**Figure 1:**
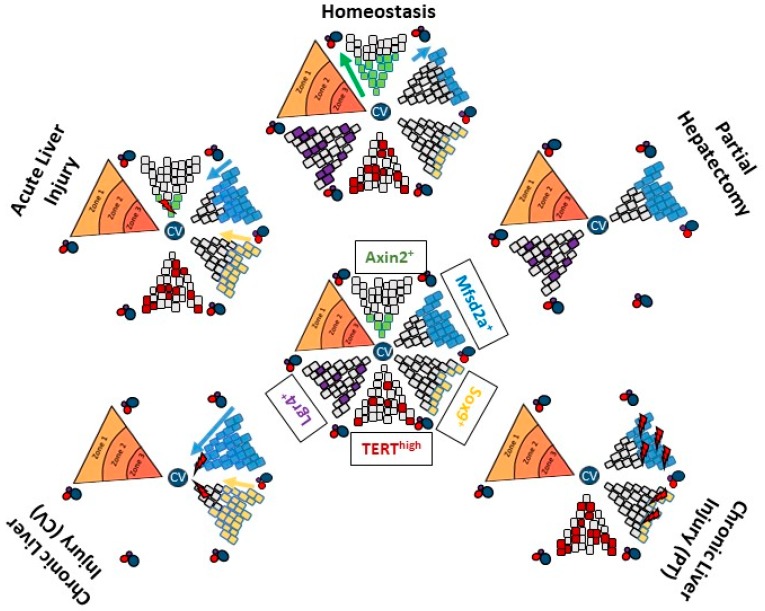
The hepatocyte subpopulations. Graphical summary of the distinct populations of hepatocytes and their contribution to parenchymal regeneration in different liver injuries. Represented at the center of the figure are the populations as traced at the beginning of any experiment. Mfsd2a: major facilitator super family domain containing 2a; Sox9: SRY-related high mobility group (HMG) box transcription factor 9; TERT: telomerase reverse transcriptase; Lgr4: Leucine-rich repeat-containing G protein-coupled receptor 4; CV: central vein; PT: portal tract.

Whether hepatocytes within the lobule all have the same regenerative capacity is still an unanswered question, however, seeing the high regenerative potential of the hepatocytes, this remains a subject of high interest. The search for the existence of different populations of hepatocytes with different regenerative capacity has recently been reinvigorated with the development of various lineage tracing tools and models, as summarized in Figure 1.

#### 2.3.1. Pericentral Hepatocytes Expressing Axin2

Recently, Wang et al. reported that a population of pericentral hepatocytes expressing Axin2repopulated the liver parenchyma during homeostasis [50], but not after PH [51]. Specifically, Axin2 is a transcriptional target of the Wnt/β-catenin pathway, which is highly activated in zone 3 hepatocytes. These hepatocytes were traced using the Axin2-CreER^T2^ mouse model. After activation of the tracing transgene, tagged hepatocytes exclusively belonged to the first cell layer around the central vein. One year after tagging, the progeny of these cells occupied up to 30% of the liver parenchyma [50]. In contradiction to this, other authors showed that the proliferative capacity of pericentral hepatocytes after PH was much lower than that of mid-zonal or periportal hepatocytes [51]. Doubts on the data generated in the Axin2-CreER^T2^ mouse model have been raised [51]: The cell cycling rate of the hepatocytes traced as Axin2 positive in a quiescent liver has been estimated from the authors’ data to be around 14 days, which does not appear to be physiologically possible, as the lifespan of a hepatocyte is about 200–400 days during homeostasis [52,53]. The proliferation rate of this tagged population could represent an artefact introduced by genetic manipulations, where the modification of the Axin2 locus may have influenced the Wnt signaling and therefore hepatocytic proliferation.

#### 2.3.2. Periportal Hepatocytes Expressing Sox9 or Mfsd2a

If the data generated by the Axin2-CreER^T2^ mouse model were correct, then during homeostasis the proportion of periportal hepatocytes should gradually decrease over time. Studies tracing periportal hepatocytes do not however fully support this proposition. Periportal hepatocytes are traced as Sox9 positive [54] and/or Mfsd2a positive hepatocytes [55]. Sox9 (SRY-related high mobility group (HMG) box transcription factor 9) is a transcription factor that expressed in hepatoblasts forming the ductal plate in the embryonic liver which give rise to the periportal hepatocytes and to cholangiocytes [56]. In the adult liver, hepatocytes expressing Sox9 are located at the limiting plate, i.e., the first layer of hepatocytes around the portal vein. They are estimated to represent less than 5% of the entire hepatocyte population. Mfsd2a (major facilitator super family domain containing 2a) labels all the hepatocytes in zone 1 and a few of the zone 2 hepatocytes [55]. The number and localization of hepatocytes traced as progeny of Sox9 periportal hepatocytes does not change with aging of the organ [54], while that of Mfsd2a decreases [55]. The proportion and lobular location of Mfsd2a^+^ hepatocytes do not change after completion of regeneration after PH, strongly supporting that periportal hepatocytes have no proliferative advantage nor disadvantage during homeostasis or regeneration. The administration of a hepatotoxicant, destroying part of the hepatocytes, is used as model of “chemical hepatectomy” to study regeneration. CCl_4_ damages hepatocytes around the central vein. After CCl_4_-induced injury, Sox9^+^ and Mfsd2a^+^ undamaged hepatocyte populations repopulate the liver parenchyma. On the contrary, when the damage is directed on the periportal area (such as in the 3.5-diethoxycarbonyl-1,4-dihydrocollidine (DDC) and the bile duct ligation (BDL) models of cholestasis), Mfsd2a and Sox9 populations are destroyed or damaged and are no longer found in the regenerated liver [54,55]. These cell tracking experiments thus support the hypothesis that any hepatocyte in the lobule is able to proliferate and regenerate the parenchyma. 

#### 2.3.3. Hepatocytes with High Expression of Telomerase Reverse Transcriptase

In 2018, a newly described population of hepatocytes with high expression of telomerase reverse transcriptase (TERT^high^ hepatocytes) has been proposed to be a determinant for hepatocytic regeneration [57]. This population represents 3–5% of all hepatocytes in a 2-month-old mouse liver. Unlike the Sox9 or the Mfsd2a hepatocytes, they are randomly distributed throughout the entire lobule. Li et al. showed that the TERT^high^ hepatocytes have a higher proliferative capacity compared to the TERT^low^ population during homeostasis, repopulating the liver up to 29.9 +/− 2.4% after 1 year. On the contrary, the TERT^low^ hepatocytes have a higher expression of metabolic enzymes. Upon acute CCl_4_ liver injury, the TERT^high^ hepatocytes increased in number and spread throughout the entire lobule. Although rare in zone 3 in a control liver, numerous TERT^high^ hepatocytes expressing the centrolobular enzyme glutamine synthase (GS) were present in the pericentral location after recovery from acute injury. These data indicate that hepatocytes out of zone 3 proliferate after the damage to repopulate the damaged area. Their progeny replaces the killed hepatocytes, adapting their zonal expression profile according to the new location they occupy. To demonstrate the importance of the TERT^high^ population in regeneration from chronic liver disease, Li et al. depleted this population specifically using the diphtheria toxin (DTA)-based adeno-associated viral system prior to cause liver injury with a DDC-containing diet. The authors showed an increase in inflammation and fibrosis, however unfortunately as DTA induced cell death in the hepatocytes and thus enhanced liver inflammation per se, interpretation on the role of TERT^high^ hepatocytes in regeneration in the chronically injured organ was precluded [57]. Selective inhibition of the proliferative capacity rather than targeted cell destruction may represent a better alternative to exploring this question. 

#### 2.3.4. Hepatocytes Expressing Lgr4

The proliferative and regenerative capacity of hepatocytes has also been evaluated, referring to the proteins involved in the Wnt/β-catenin pathway. In 2016, Planas-Paz et al. demonstrated the important role of the Leucine-rich repeat-containing G protein-coupled receptor 4 (Lgr4) [51,58]. By binding the R-spondin ligands, Lgr4 enhances Wnt/β-catenin signaling. The authors showed that Lgr4 mRNA is expressed similarly in all the hepatocytes along the porto-central axis, assigning to them the same capacity to respond to an appropriate Wnt/β-catenin stimulus. Then, they traced the Lgr4^+^ hepatocytes using a Lgr4-CreER^T2^ transgenic mouse model. After 10 months, they found clones consisting of 1–5 progeny of tagged Lgr4^+^ hepatocytes randomly distributed in the lobule. Irrespective of the lobular location, the proliferation rates were similar in all clones [51]. This supports the notion that any Lgr4 expressing hepatocytes in the lobule may contribute to the homeostatic maintenance of the liver mass. In addition to the Lgr4 receptor, pericentral hepatocytes also express the Lgr5 isoform. If expressed, the engagement of this receptor is needed to inducing proliferation: Lgr5 knockdown results in a decreased proliferation of hepatocytes around the central vein only, while the silencing of Lgr4 significantly reduces the proliferative capacity of hepatocytes within the entire lobule. 

All this shows that the conundrum of a specific population of hepatocytes involved in liver homeostasis and regeneration is unresolved. Available data support that any hepatocyte is capable of self-duplication and thereby contributing to the maintenance of the organ [51]. The preponderant contribution of a specific subpopulation appears to depend more the disease-induced zonal hepatocyte damage and on the duration and severity of the injury. For instance, the capacity of hepatocytes to regenerate the liver, evaluated after acetaminophen (APAP) or CCl_4_-induced acute liver injury, has to take into account that these toxicants destroy the population of hepatocytes around the central vein (CV). Cytochrome P450 enzymes, specifically CYP2E1 and 1A2 (which metabolize and activate these molecules), are expressed only in the hepatocytes around the CV. Central hepatocytes being destroyed, periportal hepatocytes and midlobular hepatocytes support regeneration. Very little hepatocytes adjacent to the region of cell damage were found to proliferate [59]. This has been further demonstrated using a mathematical quantitative model [60]. After inducing CCl_4_, the pericentral hepatocytes died by necrosis and the debris was cleaned up, however the endothelial cells survived, maintaining the scaffold for the lobular structure. Thus, these undamaged (midzonal and periportal) hepatocytes are able to proliferate. The mathematical quantitative model demonstrates that the dividing hepatocytes are oriented in the direction of the closest sinusoids [60]. In the same way, when periportal hepatocyte are destroyed by a pathological process, undamaged pericentral hepatocytes probably support the regeneration of the liver parenchyma. 

Recently, using single-cell analysis, Halpern et al. demonstrated that in the hepatocyte populations, around 50% of all the liver genes were significantly zonated, with clear evidence of spatial and functional correlation [61]. Thus, every single hepatocyte has a specific profile and carries out a specific function according to its exact location. The individual properties of each cell can also apply to proliferation and regeneration capacity, without a real subset of hepatocytes clustered by a specific marker. To build upon our knowledge and understand how to stimulate hepatocytic proliferation, future studies should focus on the surrounding stimuli and molecular pathways that occur around and in proliferative hepatocytes, rather than classifying them.

## 3. Ductular Reaction-Driven Parenchymal Regeneration in Chronically Injured Liver

Although hepatocyte-mediated regeneration allows for the rapid and effective functional recovery of liver mass after PH or an acute injury, this is not the case for chronic liver diseases. Chronic liver injury is damage that is built over time. It involves the continued and progressive destruction of the liver parenchymal cells concomitant to a wound healing process, leading to fibrosis and ultimately cirrhosis. The repetitive insult to the liver both stimulates hepatocytic proliferation and massively activates the HSC that become myofibroblasts. These continuously produce a large amount of ECM proteins, specifically collagen, that conclude in a disrupted liver architecture, loss of function and aberrant or exhausted hepatocytic regeneration. In such an environment, hepatocytes are not able to proliferate anymore and may thus enter into replicative senescence [62,63]. It is in this setting that the emergence of a ductular reaction (DR) is observed. This term refers to the expansion of small cells with a biliary phenotype either forming pseudo ducts within the portal mesenchyme or invading the lobular parenchyma [64]. DR is not observed following PH. For the time being, there are no available specific markers for distinguishing the putative progenitor cells or DR cells from cholangiocytes based on immunohistochemical detection. Up until now, DR cells have been isolated from rodents through centrifugation or fluorescence-activated cell sorting using a panel of antibodies. These cells were characterized as being positive for alpha-foetoprotein, albumin, cytokeratin 18 and 19 [65], or as CD45^−^CD31^−^Ter119^−^EpCAM^+^CD24^+^CD133^+^ [66] in a CDE-diet model of DR.

### 3.1. DR Cells Can Differentiate into Hepatocytes

DR is seen in virtually all human chronic liver diseases. It was from observational studies in human liver slides exhibiting small hepatocytes carrying biliary markers at the edge of the DR that the hypothesis was proposed that DR could act as a reservoir of hepatocytes that are able to contribute to parenchymal restoration [67,68,69]. Cells with an intermediate size and an immunophenotype between cholangiocytes and hepatocytes were observed in the periportal area: They were recognized as cells between 6 and 40 microns, expressing markers of both cholangiocytes (OV6, CK7) and hepatocytes (CK8, CK18, heppar-1) [68,69]. Moreover, studies on the origin of the regenerative nodules in chronic liver diseases, evaluating sequential tissue slides [67] or following a mitochondrial mutation [70], came to the conclusion that the hepatocytes in the regenerative nodules must have originated from a long lived cell or a DR cell following a maturation process. 

The first evidence that DR cells are progenitors and could differentiate into hepatocytes was provided by in vitro studies of cells sorted from liver injury animal models [71]. The in vitro behavior of these cells highly depended on the type of growth factors/cytokines they were exposed to. The hepatic growth factor (HGF) [72,73], oncostatin M (OSM) [72], the epidermal growth factor (EGF) [74] and several fibroblast growth factors (FGF) [75] promoted the commitment of DR cells towards hepatocyte lineage. An example is given by the Lgr5^+^ cells, sorted after a single CCl_4_ injection, which also expressed Sox9 but no mature hepatocyte or stellate cell markers [71]. Although morphologically resembling hepatocytes, their gene expression profile was reminiscent of biliary duct cells. It was thus proposed that this hepatocyte-like Lgr5^+^ population emerging in the injured liver derived from biliary cells [71]. When Lgr5^+^ cells were sorted by fluorescence-activated cell sorting and expanded in defined organoid culture conditions, they adopted biliary morphology and features. While exposure to the Notch inhibitor [71], dexamethasone [76] for the inhibition of the TGF-β signaling, fibroblast growth factor 19 (FGF19) [77] and bone morphogenetic protein 7 (BMP7) [78] all reduced cell growth and fostered hepatocyte differentiation [71,79]. 

Other indirect evidence (such as similar mitochondrial mutation in DR and small new hepatocytes [70]) corroborates the idea of DR cells having potential to differentiate into hepatocytes. Nevertheless, these observations did not refute the alternative proposition that both intermediate hepatocytes and DR cells stemmed from hepatocyte metaplasia. 

**Figure 2:**
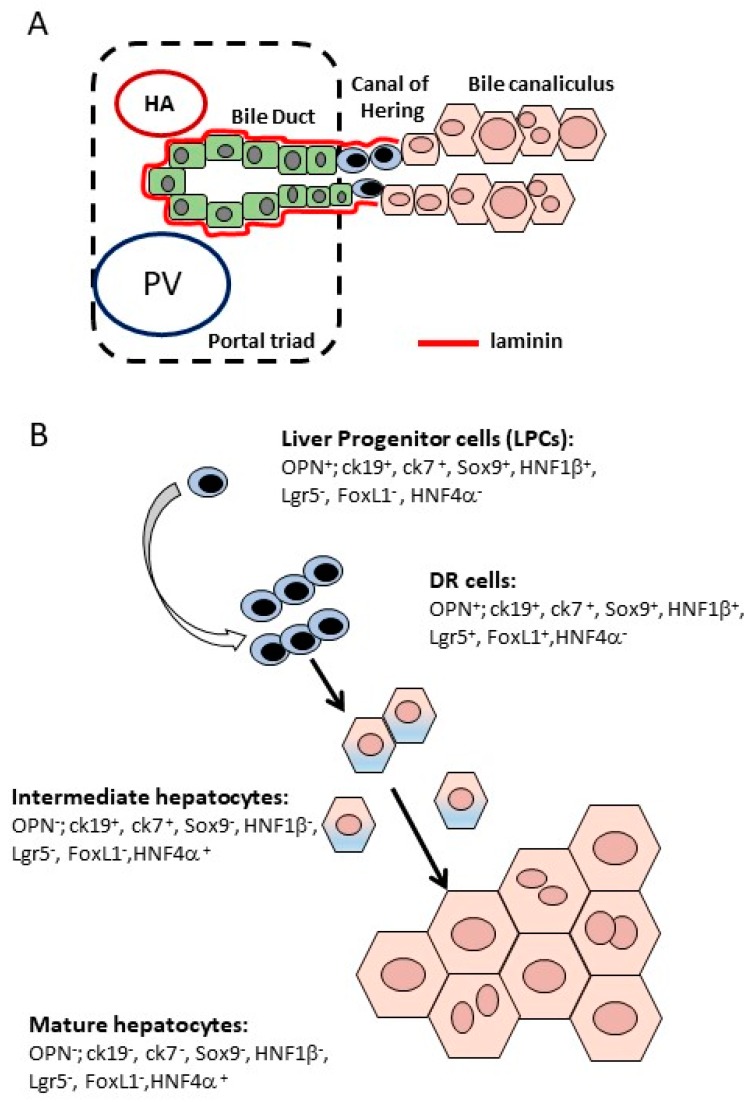
The liver progenitor cells (LPCs) and ductular reaction (DR). (**A**) Localization of LPCs within the liver; (**B**) dynamics of the differentiation process of DR cells: During injury, DR starts to emerge; DR cells will differentiate into hepatocytes through intermediate steps.

In the last few decades, researchers used several genetic lineage-tracing mouse models to study the origin and fate of DR cells. Work from our laboratory demonstrated that the embryonic ductal plate cells generated in the adult liver cholangiocytes line the most proximal segments of the bile ducts and the canal of Hering, as well as some periportal hepatocytes [56]. The DR emerging in the injured liver was also derived from the ductal plate cells [56]. The laboratory also provided the first in vivo evidence that biliary cells do differentiate into hepatocytes in some disease conditions [80]. This result was obtained by tracing OPN^+^ biliary cells [80] and has been confirmed in several other mouse models tracing CK19^+^ [66,81,82] or HFN1β^+^ cholangiocytes [83,84]. More recently, the contribution of DR cells to parenchyma regeneration was also shown in a model of thio-acethamide-induced chronic disease, where 7% of the hepatocytes were demonstrated to be of biliary origin [82]. In our experiments, biliary-derived hepatocytes were found at the proximal edge of the DR during injury and in place where the DR was in injury recovery samples. This supports the hypothesis that the DR cells are the ones that undergo hepatocyte differentiation [80]. Further independent studies traced forkhead box1 (Foxl1), a member of the forkhead winged transcription factor family, and Lgr5, as already described. Both Foxl1 and Lgr5 are not detected in a healthy liver but are found in a injured liver in DR cells and in some cholangiocytes [71,85]. The authors of these studies propose that Foxl1 and Lgr5 label proliferative subpopulations emanating from a biliary progenitor, the so-called transit amplifying cells, and both studies arrived at the conclusion that DR cells can differentiate into hepatocytes (Figure 2). Conversely, existing literature also supports the notion of hepatocyte to biliary phenotypic trans-differentiation. Using cholangiocyte or hepatocyte cell tracking tools, evidence was found of a trans-differentiation of hepatocytes in DR in animals undergoing cholestatic liver injury, such as DDC and BDL [86,87,88]. These hepatocyte-derived biliary cells were also shown to re-differentiate into hepatocytes after the injury was reversed [86,89,90,91]. 

### 3.2. Molecular Signals Involved in DR Expansion and Differentiation

The data strongly support the potential of DR cells in differentiating into hepatocytes. All of these studies also revealed ligands and signaling events that influence DR proliferation and differentiation in mice. Some of them have already been described in detail in previous reviews [92,93]. To cite some of them, the inflammatory response of TNF and fibroblast growth factor-inducible molecule 14 (Fn14) signaling are required for DR expansion. Indeed, knocking down TNF receptor 1 [94,95] or using an antibody against a TNF-like weak inducer of apoptosis (TWEAK) [96], leads to a drastic decrease in the DR response. Molecules that activate these pathways are produced by inflammatory cells which are found in the DR niche [97]. Additionally, HGF or the c-MET-signaling pathway affected the proliferation of the DR cells. Moreover, this pathway is also important for hepatic differentiation of the DR. Quantitative analysis of c-MET deficient mice fed with DDC revealed a decline in the percentage of DR cells, as well as of DR-derived hepatocytes (identified as A6^+^/HNF4α^+^ cells) compared to the control mice [98]. FGF7 (produced by mesenchymal cells) has also been identified as a critical regulator of DR expansion [99]. Recently, data indicated that brain-expressed X-linked 1 (BEX1), a modulator of intracellular signaling expressed in the DR cells in CDE-injured livers, plays a pivotal role in DR activation and expansion as well [100]. Hepatocytic and cholangiocytic differentiations are driven by different signals. The activation of the Notch pathway, mediated by Jagged-1 which is secreted by myofibroblasts, is required to maintain DR in a biliary phenotype and to complete biliary differentiation [101,102]. The Hedgehog and Hippo/YAP pathways also play a role in biliary differentiation, interacting with the Notch pathway [103,104]. For hepatocyte differentiation, Numb, a direct transcriptional target of the Wnt signaling pathway, will cause an inhibition of the Notch pathway and an activation of the Wnt pathway. Wnt3a (which is secreted by macrophages in the DR niche) is particularly important in this process [101]. The Hedgehog pathway is also involved in the macrophage recruitment process [105], and thus in the process of hepatocyte differentiation [106]. Furthermore, spatially intimate correlation of DR with the extracellular matrix has been described, identifying matrix components such as the laminin-basal membrane as contributors of DR biliary phenotype maintenance [56,80,97]. Escaping from this membrane promotes the hepatocytic differentiation of DR cells [80]. Finally, signals emanating from the sensing of bile modification (flow and composition) should also be considered as potential DR modulators. Tauro-conjugated BAs were shown to promote cholangiocyte proliferation, and more recently, to initiate the proliferation and biliary differentiation of DR cells [107,108]. Taurocholate stimulates cell growth by inducing the transactivation of the EGF receptor in cholangiocytes, via a TGF-α and matrix metalloproteinase-dependent mechanism, similar to what is reported in hepatocytes [109,110].

All these data support the notion that complex intricate signals from the surrounding niche modulate DR proliferation and differentiation. Responding to injury-specific signals, DR can be pushed to differentiate into hepatocytes.

### 3.3. Significance of DR-Mediated Parenchymal Regeneration

To evaluate the significance of hepatocyte generation from DR cells for parenchymal regeneration during injury, Shin et al. conditionally ablated the Foxl1+ cells in the CDE liver injury mouse model. They showed that such a deletion prevented the resolution of hepatic steatosis [85]. Thus, they intriguingly concluded that as the prevention of DR-driven regeneration worsens the liver condition, the “Foxl1+ DR cells are required for the development of hepatocytes after CDE-induced liver injury” [85]. Another study that was based on single Lgr5+ cells isolated from a damaged mouse liver clonally expanded as organoids in a R-spondin1-based culture medium and were transplanted into fumarylacetoacetate hydrolase deficient (FAH^−/−^) mice, a model for tyrosinemia type I liver disease in which liver failure occurs unless a treatment is applied [71]. The authors showed the engraftment of Lgr5^+^ DR cells and their differentiation into mature HFN4α^+^/CK19^−^ hepatocytes, amounting to 1% of the liver parenchyma in a small proportion of transplanted animals. Furthermore, they observed an increased rate of survival of mice transplanted with the Lgr5^+^ cells compared to the non-transplanted group [71]. This supports the hypothesis that transplanted Lgr5^+^ cells, although poorly repopulating the liver, contribute to liver function. Other experiments in which DR cells were isolated from CDE or DDC-injured livers and transplanted into mice with deficient liver function (either AhCre^+^, Mdm2^flox/flox^ or in FAH^−/−^ mice) confirmed the capacity of such cells to differentiate into mature hepatocytes. However, those studies did not report whether this was sufficient to rescue liver function and ensure the survival of recipient mice [66,111]. 

Although all the studies mentioned above confirm the potential for hepatocyte differentiation in biliary/DR cells, the number of biliary/DR-derived hepatocytes generated in vivo during injury is low (<7%) [80,82,83,84]. The observations in the transplantation experiments or after Foxl1 ablation fail to prove that the DR/biliary compartment significantly contributes to functional parenchymal reconstitution. However, rather than reflecting a lack of functional importance of DR, these results could suggest that DR may have physiological functions other than parenchymal reconstitution (Clerbaux et al., in preparation). This discussion is beyond the topic of this review which focuses on parenchymal restoration.

## 4. Boosting Hepatocyte or DR-Mediated Parenchymal Regeneration as a Potential Alternative Strategy to Alleviate Liver Insufficiency

Regeneration recreates a well-sized functioning organ. When the process is abrogated or inefficient, liver failure ensues. Currently, liver transplantation is the only valid strategy for treating acute liver failure and the end-stages of chronic liver insufficiency. Cell transplantation and the stimulation of the endogenous regenerative process are attractive alternative therapeutic strategies.

### 4.1. Stimulate in vivo DR-Mediated Parenchymal Regeneration?

Despite our growing knowledge on liver regeneration, we failed to stimulate hepatocyte proliferation beyond a physiological response. Hence, therapeutic means to enhance hepatocyte proliferation are not available. The stimulation of DR-driven regeneration may still hold therapeutic potential. Based on the identified signals promoting DR expansion and the differentiation into hepatocytes, in vivo stimulation of endogenous DR cells could amplify DR-driven regeneration and thereby provide new functional hepatocytes to a diseased liver. First, the inflammatory signaling pathways could be manipulated. For instance, the depletion of macrophages during recovery, worsening the wound healing process compared to the control, with a persistence of the collagen and myofibroblasts [112]. Similarly, CD11b-knockout mice monocyte-derived (infiltrating) macrophages decreased after PH. These mice showed a lower liver mass regeneration and an increase in mortality rate [113]. Thus, both liver-residing and infiltrating macrophages are important for liver regeneration. Importantly, when in the DR niche, they secrete Wnt3a which activates the Wnt/β-catenin pathway, the activation of which is related to the DR hepatocyte specification [101]. Another interesting approach could be the administration of growth factors, such as HGF, an important mitogen for angiogenesis and hepatocytic proliferation [114,115]. As it secretes growth factors as well as forming new vasculature necessary to promote and orient hepatocyte proliferation, the endothelium represents an attractive candidate for manipulation, via angiogenic agents for example [116]. Besides, the modulation of BA signaling is another possible strategy for enhancing regeneration [117]. 

### 4.2. Pitfalls of Liver Regenerating Therapies

Importantly, in order to succeed, the in vivo stimulation of endogenous cells has to (both and in parallel) enhance parenchymal regeneration and promote fibrosis resolution. Fibrosis associates with DR: ECM deposition precedes the development of the DR [97], suggesting early ECM deposition is independent from DR cells. Stimulation of the DR was associated with an increase of liver fibrosis [118], conversely, DR inhibition reduced the amount of ECM [119]. Also, ECM influences the DR fate as the suppression of laminin expression drives a higher number of DR-derived hepatocytes. DR cells are also able to secrete multiple soluble pro-fibrogenic factors acting on HSC and myofibroblasts [107,120]. Their expression of integrin αvβ6 is correlated with fibrosis progression in human models of biliary fibrosis [121]. Selective targeting of this integrin in mouse models of chronic biliary injury inhibits the DR/progenitor response and protects against liver fibrosis [122]. Thus, it is still not clear whether the tight association of DR-fibrosis is beneficial for DR-driven regeneration, or whether DR reinforces fibrosis. Understanding the signals that balance regeneration and fibrosis is thus essential to obtaining a less friendly fibrotic environment, but at the same time a pro-regenerative one.

Amplifying proliferative signals, in hepatocyte or DR cells, as in any other cell type, may bear a dark side: The initiation of carcinogenesis. Indeed, as DR cells possess self-renewing and proliferative capacities, their presence often is accompanied by cancer progression [123]. They have been proposed to contain a population of cancer stem cells [124]. Alternatively, the progenitor signature which is observed in tumors presenting the worse prognosis might reflect the plasticity of tumor cells with a dedifferentiation of the hepatocytes [125]. The fact that many liver tumors arise during cirrhosis, when hepatocyte senescence triggers the activation of DR, causes confusion. Thus, the cell of origin as well as the mechanisms involved in liver cancer have yet to be precisely identified before manipulating the DR cell compartment for therapeutic purposes.

## 5. Conclusions

Liver regeneration is a complex process involving intricate signals to and from different cell types. Clearly, the mechanisms are not totally untangled, yet significant progress identifying regenerative injury-specific signals has been made, opening new avenues for boosting liver parenchymal restoration. Hepatocytes represent the main actors in parenchymal regeneration in both rodent models and humans. The recent regenerative heterogeneity observed in hepatocytes suggests that tailoring their local microenvironment may be of benefit for regeneration. To understand how to stimulate hepatocyte proliferation, future studies should focus on deciphering the surrounding stimuli and molecular pathways that occur around and in proliferative hepatocytes. In the case of replicative exhaustion, DR cells emerge in the diseased liver and may represent a rescue compartment to mediate parenchymal restoration. Although a tremendous amount of work is still needed to integrate all the signals controlling the process and the physiological significance of DR-driven regeneration, it is reasonable to predict, based on available data, that in vivo simulation of the surrounding DR niche would in the near future represent a (complementary) therapy to boost parenchymal reconstitution.
